# Atrial Fibrillation Induces Sarcomere Remodeling, Enhanced Sarcomere Contractility, and Loss of Atrial Identity

**DOI:** 10.21203/rs.3.rs-6422874/v1

**Published:** 2025-04-23

**Authors:** Jonathan Kirk, Christine Delligatti, Ilhan Gokhan, Parth Desai, Rosie Barrows, Ahmed Zied, Geena Fritzmann, Seby Edassery, David Barefield, Steven Niederer, Stuart Campbell, Michaela Door

**Affiliations:** Loyola University Chicago Stritch School of Medicine; Loyola University Chicago Stritch School of Medicine; Yale University; Loyola University Chicago Stritch School of Medicine; Imperial College London; Loyola University Chicago Stritch School of Medicine; Loyola University Chicago Stritch School of Medicine; Loyola University Chicago Stritch School of Medicine; Loyola University Chicago Stritch School of Medicine; Imperial College London; Yale University; Loyola University Chicago Stritch School of Medicine

## Abstract

Atrial fibrillation (AF) is the most common arrhythmia, with few treatment options. To discover novel pathways, we performed mass spectrometry (MS) on atrial tissue from patients in Sinus Rhythm or with AF without heart failure. We identified changes in canonical AF pathways, although surprisingly, contractile proteins and specifically a loss of atrial isoforms. Functional remodeling was confirmed in AF cardiomyocytes, revealing increased contractility compared to SR. We performed MS analysis of human atrial and ventricular tissue and found that ~1/3 of proteomic remodeling in AF was associated with chamber identity. Using atrial hiPSC-CM Engineered Heart Tissues to model AF, we replicated proteomic and contractile remodeling observed in human tissue, indicating mechano-sensing likely drives these effects. Lastly, an integrative patient simulation suggests this cellular remodeling is likely maladaptive. Together, these results reveal a novel role for sarcomere remodeling and a loss of atrial identity in AF, representing potential new therapeutic targets.

## Introduction

Atrial fibrillation (AF) is the most common cardiac arrhythmia, affecting over 50 million people globally with upwards of 13% of US AF cases remaining undiagnosed (1). Furthermore, the prevalence of AF has steadily increased over past decades and is expected to continue to grow by as much as 60% by 2050 (2). Patients with AF experience at least a 4 to 5-fold increase in the risk of stroke, and around 1/3 of all strokes are caused by AF (3). Current therapies target the electrophysiological substrate for AF, as this is the best understood mechanism (4–6). These clinical approaches include rate control, anti-arrhythmogenic drugs (7), catheter ablation (8), and the Maze procedure (9) (a surgical approach in which a pattern of scar tissue is created by the surgeon to block abnormal electrical rhythms). However, these therapies have success rates as low as 50%, permanently damage cardiac tissue, or are paradoxically likely to be pro-arrhythmic (8–10). Thus, there is a pressing need for new therapeutics to treat AF.

Substantial foundational science work has been done to understand the mechanisms driving the development and persistence of AF (11–16). Indeed, promising lines of investigation include ion channel remodeling, extracellular matrix and fibrosis, inflammasome activation, metabolic remodeling, and others. However, these advancements have not yet been successfully translated into clinical therapies; in fact, between 1990 and 2019 there was no significant change in age-standardized AF-related mortality as seen by a Global Burden of Disease study (17). We hypothesize key pathways remain to be elucidated to develop more effective therapeutics for these patients.

One major challenge facing AF foundational research is availability of appropriate models, as most animal models of AF have substantial caveats to their translatability to humans (18). For research groups studying human AF atrial tissue, these banked tissues often come from transplant recipients, adding variables that may confound interpretation of data. Other work utilizes left atrial appendage (LAA) tissue (19) that is frequently excised during various procedures due to the risk of formation of clots within the LAA. However, the LAA has many distinct features compared to the main atrial wall and may not be representative of the whole atria in healthy or diseased conditions. Thus, our first goal in this study was to perform unbiased high resolution discovery mass spectrometry on non-failing left atrial main wall tissue from normal SR patients and those with paroxysmal AF.

We performed proteomic analysis of atrial samples from patients in SR and those with AF and discovered substantial sarcomere remodeling, primarily a decrease in atrial specific isoforms and increased levels of ventricular isoforms. Indeed, this pattern was observed across the entire cardiac proteome, as when we performed MS analysis of atrial and ventricular human samples, approximately one third of all proteins altered by AF were also differentially expressed at the chamber level. Recently, it has been discovered that variants in sarcomeric proteins are associated with AF (20–25), but almost no work has been done examining sarcomere function in AF. Here we report a novel role for sarcomere proteomic and functional remodeling in AF, including a general loss of atrial identity. These findings reveal a novel therapeutic target for this growing disease.

## Results

### Atrial Fibrillation Causes Sarcomere Remodeling and Loss of Atrial Identity

To identify novel pathways involved in atrial fibrillation (AF), we began by performing a comprehensive assessment of the proteomic landscape in main wall left atrial tissue samples from non-failing rejected donor hearts in normal sinus rhythm (SR) or with a history of paroxysmal AF. The demographics for these patients (Table 1) are not significantly different between the groups.

**Figure 1:**
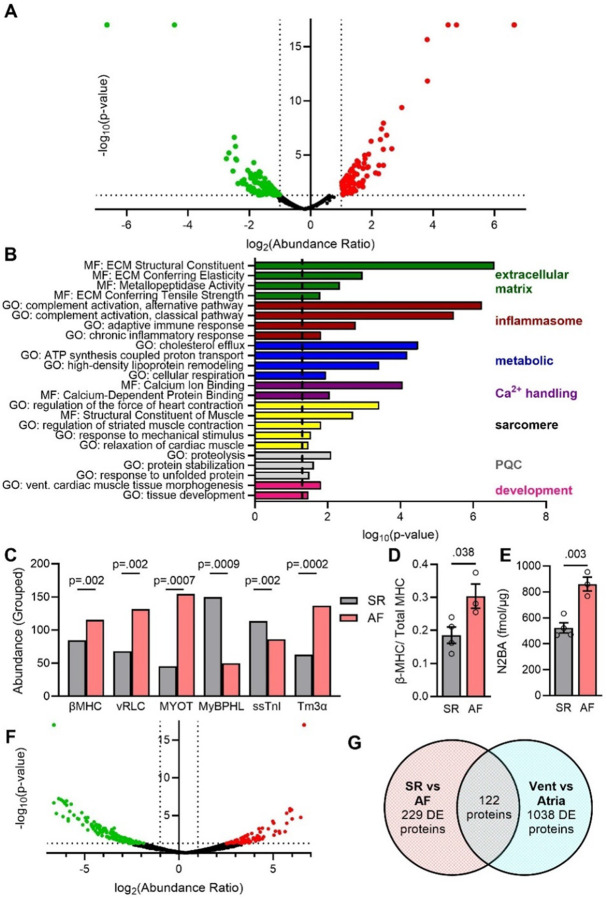
Atrial Fibrillation Induces Proteomic Remodeling, Resulting in a Loss of Atrial Identity. **(A)** Volcano plot from label-free mass spectrometry (MS) analysis of left atrial tissue homogenates from non-failing rejected donor hearts in Sinus Rhythm (SR, n=3) and with paroxysmal atrial fibrillation (AF, n=3), each point representing a protein that was significantly increased (red), significantly decreased (green), or unchanged in the AF group compared to NF. **(B)** Selected significantly enriched Molecular Function (MF) and Gene Ontology (GO) classifications from pathway analysis of differentially expressed (DE) proteins in panel A. **(C)** Abundance ratios from mass spectrometry analysis of SR and AF samples, for selected atrial- or ventricular-enriched sarcomeric proteins. **(D)** Multiple Reaction Monitoring (MRM) MS absolute quantification for β-myosin heavy chain (β-MHC) and the **(E)** N2BA titin isoform. **(F)** Volcano plot generated from label-free MS analysis of left atrial and left ventricular tissue homogenates from non-failing rejected donor hearts in SR. **(G)** Venn diagram showing the overlap in DE proteins identified in the SR vs AF (panel A) and Ventricular vs Atrial (panel F) MS experiments. Statistics by Proteome Discoverer and two-tailed t-test.

Using whole tissue homogenates from flash frozen LA tissue from SR (n = 3) and AF (n = 3) patients, high resolution MS/MS analysis identified a total of 3,610 proteins (**Supp. Table 1**). Comparing the SR and AF groups using label-free area-under-the-curve quantification, 359 proteins were differentially expressed, 222 proteins were significantly downregulated, and 136 were significantly upregulated (p<0.05, 1<log_2_(FC)<−1) in the AF samples (Fig. 1A). To understand what pathways were disrupted in AF in the absence of heart failure, we performed bioinformatics analysis of enriched functional annotations among all differentially expressed proteins using DAVID (2021 update) (26, 27). Overall, we identified 71 dysregulated biological process (BP) pathways and 48 dysregulated molecular function (MF) pathways (Gene Ontology (GO) analysis; p<0.05, **Supp. Table 2**). Among these, we identified many expected affected pathways that are the target of substantial foundational science work and represent known hallmarks of human AF as studied across patient populations (11, 12, 28–32) (Fig. 1B). These pathways include extracellular matrix (green), inflammation (red), metabolic (blue), and calcium handling (purple). These results provide a “positive control” for the proteomic analysis, demonstrating that the findings reproduce previously identified aspects of AF.

Unexpectedly, we also found pathways associated with the cardiac sarcomere significantly dysregulated in AF. This included “regulation of the force of heart contraction”, “regulation of striated muscle contraction”, and “relaxation of cardiac muscle” (Fig. 1B, yellow). When examining the individual sarcomere proteins that were differentially expressed in the AF samples, we found increased levels of ventricular isoforms (β-Myosin heavy chain, ventricular regulatory light chain), and decreased levels of atrial isoforms (myosin binding protein H-like, ssTnI) (Fig. 1C). Typically, β-MHC is enriched in the human ventricle, while α-MHC is enriched in the human atria (33). Given the critical nature of myosin isoforms regarding contractile function, we further validated this by gel electrophoresis (**Fig. S1**), and with a targeted absolute quantification mass spectrometry approach known as Multiple Reaction Monitoring (MRM, assay development shown in **Fig. S2**) which showed a 50% increase in βMHC expression in the AF atria (Fig. 1D). We also developed and utilized an MRM assay for the titin isoforms N2B and N2BA, which are also critical for function and are not easily measured in discovery proteomics. We found the compliant N2BA titin isoform was increased in AF (Fig. 1E), suggesting chamber dilation that is frequently observed in AF patients.

We noted a pattern of decreased atrial isoform expression and increased ventricular isoform expression. We thus wanted to determine whether this was a broad proteomic signature associated with atrial fibrillation. We performed a mass spectrometry experiment comparing atrial and ventricular tissue in non-failing SR patients (paired samples from the same heart) to broadly catalogue all chamber-level proteomic changes. Unsurprisingly, there were many differences: 1,160 proteins were differentially expressed between atria and ventricles (Fig. 1F, **Supp. Table 1**). Furthermore, 122 proteins overlapped when comparing the two DE protein lists (SR vs AF and Atria vs Ventricles) (Fig. 1G). Therefore, nearly 1/3 of proteins altered by AF (122/359) were associated with chamber identity – suggesting a general, not sarcomere-specific, loss of atrial identity in AF.

### AF Results in Increased Sarcomere Function

**Figure 2:**
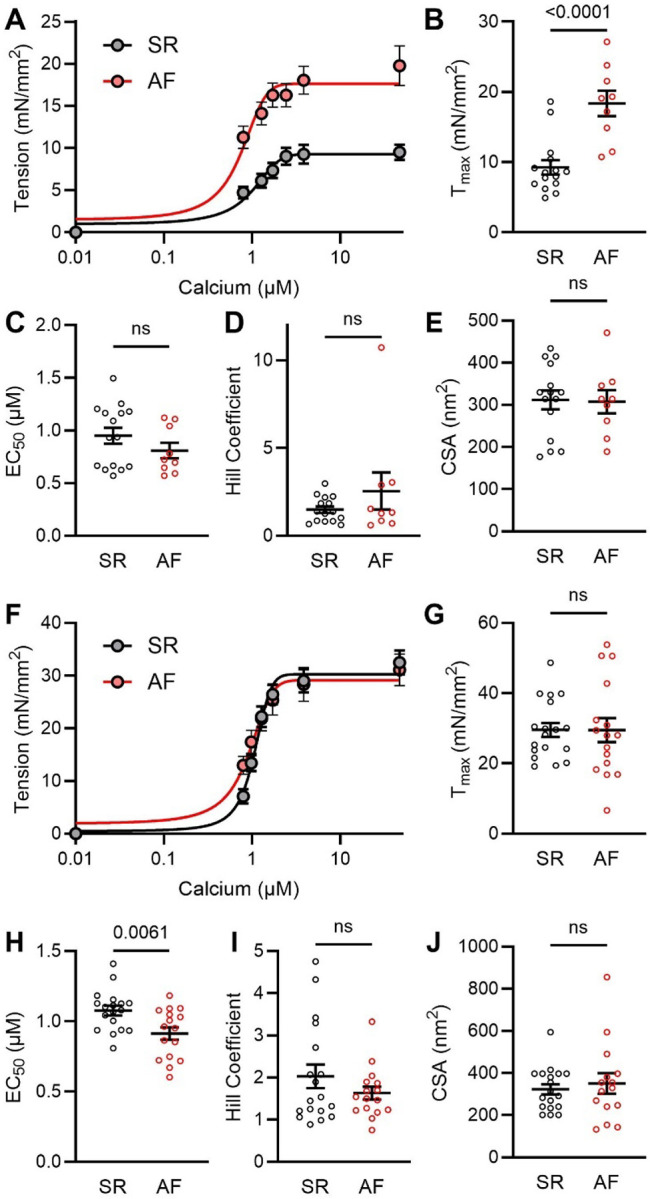
AF Increases Atrial Sarcomere Function. **(A)** Mean tension as a function of calcium concentration and fitted Hill equation curves for skinned atrial cardiomyocytes from SR and AF samples. **(B)** Summary data for calculated maximal calcium activated tension (T_max_), **(C)** Calcium sensitivity (EC_50_), and **(D)** Hill Coefficient corresponding to the tension-calcium graph in panel A. **(E)** There were no differences in the Cross-Sectional Area (CSA) for the SR vs. AF cells. **(F)** Mean tension as a function of calcium concentration and fitted Hill equation curves for skinned ventricular cardiomyocytes from SR and AF samples. **(G-J)** T_max_, EC_50_, Hill Coefficient, and CSA for corresponding tension-calcium graph in panel F. All data presented as mean ± SEM and analyzed by two-tailed *t*-test.

Proteomic analysis revealed an unexpected sarcomere and chamber remodeling associated with AF, specifically reducing atrial-associated isoforms and increasing ventricular ones. While the sarcomere remains understudied in the atria, there are several reported differences between atrial and ventricular sarcomere function (34). We thus assessed contractile function by tension-calcium skinned myocyte experiments in the left atria of non-failing SR and AF patients (Fig. 2A). Interestingly, there was a significant increase in maximal calcium-activated tension (T_max_) in AF atrial cardiomyocytes (Fig. 2B) compared to SR cardiomyocytes, with no differences in calcium sensitivity (EC_50_; calcium concentration required to achieve half-maximal tension), Hill Coefficient, or cardiomyocyte cross-sectional area (CSA, Fig. 2C–E).

We also measured tension-calcium relationships in ventricular cardiomyocytes from these same patients (Fig. 2F). There was no difference in T_max_ between the groups (Fig. 2G). This was expected, as these hearts are not in heart failure (reduced ventricular T_max_ is a hallmark of heart failure with reduced ejection fraction (35)). Ventricular AF cells also displayed a slight increase in calcium sensitivity (decreased EC_50_) (Fig. 2H), but no change in Hill Coefficient or CSA (Fig. 2I, J). The ventricular cardiomyocytes generated significantly more tension than the atrial ones in SR. However, the increase in tension associated with AF brought the atrial contractility much closer to the ventricular cardiomyocytes, indicating functional remodeling that agrees with the proteomic remodeling observed.

**Figure 3:**
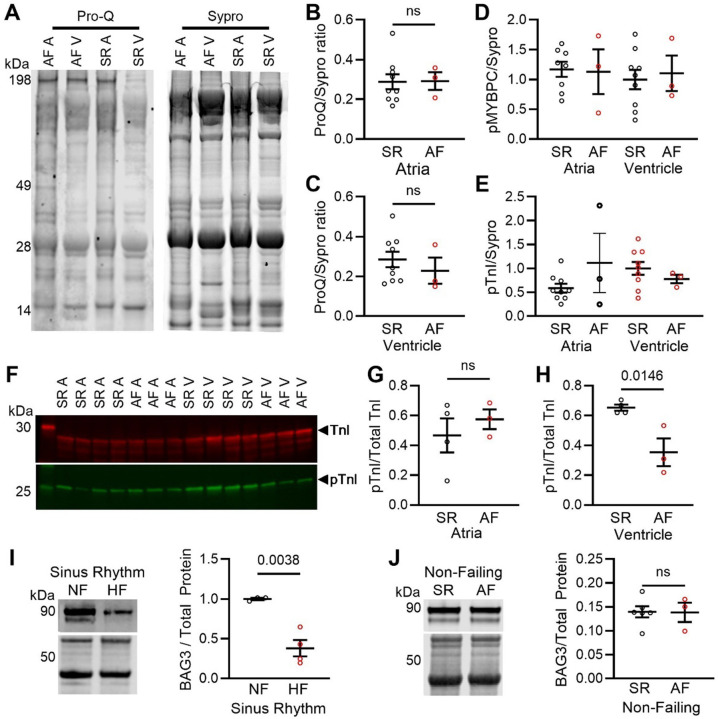
Regulation of sarcomere function is not altered in AF atrial cardiomyocytes. **(A)** ProQ (phosphorylation) and Sypro (total protein) stain of myofilament-enriched SR and AF samples **(B-E)**Quantification of panel A showing total phosphorylation in the atria (panel B) and ventricles (panel C), phosphorylation of cardiac myosin binding protein C (cMyBPC) (panel D) and TnI (panel E). **(F)** Western blot for total cardiac troponin I (cTnI) and p-cTnI in human SR (n=4) and AF (n=3), and quantification for **(G)** atrial and **(H)** ventricular samples. **(I)**Western blot and quantification for BAG3 in human atrial samples from sinus rhythm non-failing (NF) and non-ischemic Heart Failure (HF) samples. **(J)**Western blot and quantification for BAG3 in Non-Failing SR and AF samples. All data presented as mean ± SEM and analyzed by two-tailed *t*-test.

We next determined whether additional mechanisms beyond sarcomere protein isoforms may be impacting AF cell function. Sarcomere function can be strongly regulated by protein phosphorylation, which is commonly dysregulated in disease (36, 37). We first examined broad changes in phosphorylation using a ProQ (phospho) and Sypro (total protein) gel stain in myofilament enriched SR and AF samples (Fig. 3A). There were no changes in total sarcomere phosphorylation in atrial or ventricular tissue (Fig. 3B, C). We also examined phosphorylation of two proteins known to be powerful regulators of sarcomere function (36, 38): cardiac Myosin binding protein C (cMyBPC) (Fig. 3D) and cardiac troponin I (cTnI). While there were no differences in cMyBPC phosphorylation (Fig. 3D), there was a trending difference in cTnI phosphorylation (Fig. 3E). To more carefully examine cTnI phosphorylation, , we used an antibody against the S23/24 phosphorylation sites that are targets of Protein Kinase A (37). While there was no change in cTnI phosphorylation in the atrial groups (Fig. 3F, G), cTnI phosphorylation was decreased in the AF ventricles (Fig. 3H), which would result in the calcium sensitivity increase we observed (Fig. 2H). There were no changes in the total levels of either protein (**Fig. S3, S4**).

### BAG3 Levels in AF Patients Do Not Suggest Heart Failure-Like Phenotype

Previous studies of atrial sarcomere function identified a decrease in T_max_ associated with AF, in contrast to our findings here of increased sarcomere contractility in AF. While it has been shown that β-MHC is associated with greater force production (39), and thus our observed functional and proteomic data align, we wanted to identify a possible explanation for the disagreement with the literature. It has been previously shown that the atria of AF patients exhibit a heart failure (HF)-like phenotype marked by reduced cardiomyocyte contractility and increased levels of β-MHC (33, 40). In HF, impaired contractility is partially explained by a loss of sarcomere protein quality control regulated by the co-chaperone Bcl2-Associated Athanogene 3 (BAG3).

Thus, we measured BAG3 levels to investigate whether this could help explain why decreased T_max_ could be observed in some AF patients. We show for the first time in atria that SR HF patients have a significant decrease in BAG3, similar to previous observations in ventricles (Fig. 3I, ~62% decrease). However, BAG3 levels were unchanged in the atria of non-failing AF patients compared to SR patients (Fig. 3J). This suggests that in the complicating presence of HF or cardiomyopathy, decreased T_max_ may be observed in AF due to a loss of sarcomere PQC. As BAG3 levels were not dysregulated in the non-failing AF samples used in this study, we observed increased contractile function associated with increased ventricular protein isoform expression.

### Sarcomere Remodeling in AF May Result from Loss of Atrial Shortening

We next sought to further understand the proteomic and functional remodeling that occurs in the sarcomere with AF. The sarcomere is strongly regulated by internal and external mechanical forces. So, we hypothesized a mechanical cue might be responsible for our observed effects from AF. In the contraction cycle of AF atria, there is phase in which cell shortening is impaired due to overlapping stimuli from the fibrillating atria, causing the myocytes themselves to remain nearly isometric, resulting in a loss of the “boost” function (atrial kick) at the end of ventricular filling (41).

**Figure 4:**
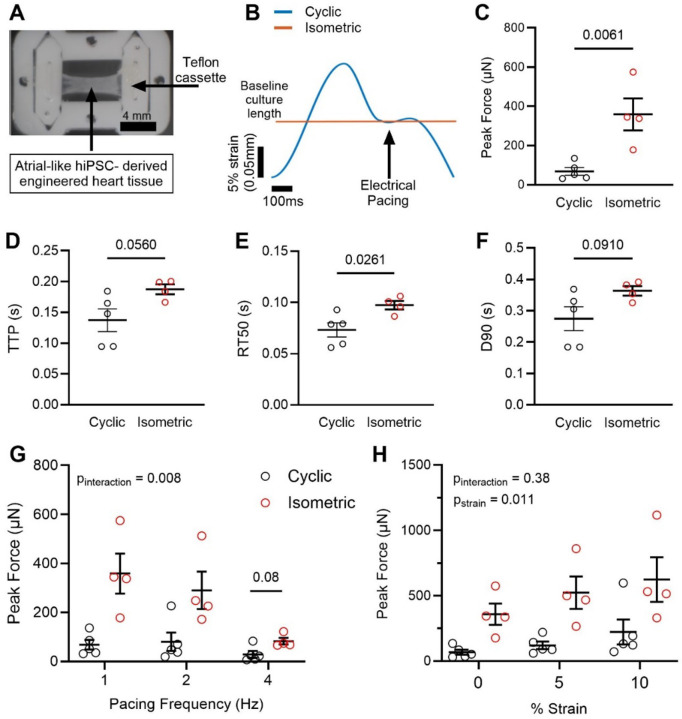
Human iPSC-CM Engineered Heart Tissues (EHT) Subjected to Isometric Strain Display Increased Force Production. **(A)** Diagram of EHT inside of Teflon cassette. **(B)** Pacing protocol for EHTs in culture for cyclic and isometric strain patterns. **(C-F)** Summary data for Peak Force (panel C), Time to Peak (TTP, Panel D), Relaxation Time to 50% peak (RT50, Panel E), and twitch duration from stimulation to 90% relaxation (D90, Panel F), in cyclic (n=5) and isometric (n=6) groups. **(G)** Effect of acute change in pacing frequency (1–4 Hz) on cyclic and isometric EHTs. **(H)** Effect of acute changes in strain (up to 10% culture length) on cyclic and isometric EHTs. All data presented as mean ± SEM, panels C-F analyzed by two-tailed *t*-test and panels G-H analyzed by two-way repeated measures ANOVA.

To model this mechanical stress associated with AF, we used engineered heart tissue (EHT). EHTs were formed from human induced pluripotent stem cells (hiPSCs) differentiated into atrial-like cardiomyocytes (hiPSC-aCMs). hiPSC-aCMs were formed into EHTs by seeding them into decellularized porcine myocardial slices, the ends of which were mounted in Teflon clips (Fig. 4A). Using a dynamic-culture bioreactor, the EHTs received either cyclic strain meant to mimic the contraction cycle of the atrium under SR (Cyclic) or were held isometrically to simulate the shortening deficit present in AF myocytes (Isometric) as shown in Fig. 4B, **S5**. Function was then measured in both groups under the same conditions (isometric, 36 °C, 1 Hz pacing). This model recapitulated the human skinned myocyte data, exhibiting enhanced Peak Force in the Isometric group (Fig. 4C). We also observed changes to the kinetics of the force waveform, including a trend toward slowed force development (increased Time To Peak, TTP, p=0.056, Fig. 4D) and slowed relaxation (RT50, Fig. 4E). While the β-MHC isoform generates more force than α-MHC, it also has slower kinetics, so this is not unexpected. The total duration of the twitch, from stimulation to 90% relaxation, also showed a trending increase (Fig. 4F).

We next acutely exposed the EHTs to stresses associated with AF, including high beat rate and pathological stretch (20, 34). There was a significant interaction between strain model (Cyclic/Isometric) and pacing frequency: isometrically-treated tissues had a steeper negative force-frequency relationship (Fig. 4G). Even at 4 Hz stimulation, peak force trended higher in isometrically-treated tissues than the cyclic control (p = 0.08). However, the cyclically-treated tissues produced a greater fold-change in force production despite producing lower absolute force. This may indicate the normal mechanisms that hold myosin heads in reserve at shorter sarcomere lengths are diminished in AF, such that when stretched to longer lengths, there is a smaller reserve pool to recruit from and hence diminished length-dependent activation.

**Figure 5:**
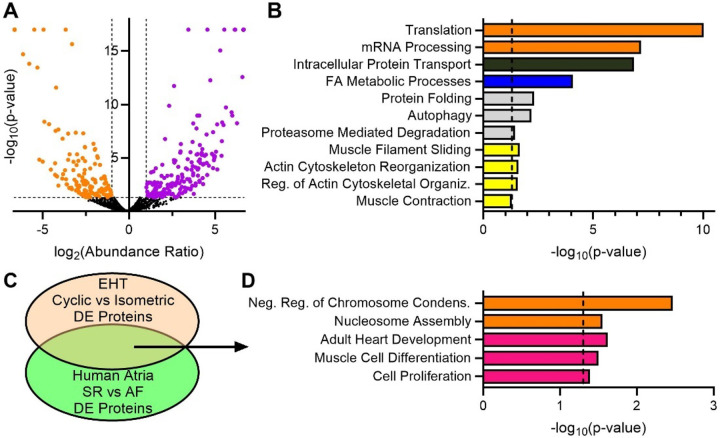
Proteomic analysis of hiPSC-CM EHTs Supports Sarcomere Remodeling and Altered Transcription Program. **(A)** Volcano plot of hiPSC-CM EHTs from cyclic (n=5) and isometric (n=5) groups, showing upregulated differentially expressed proteins (purple) and downregulated DE proteins (orange) in the isometric group. **(B)** Selected significant results from gene ontology pathway analysis of DE proteins in isometric/cyclic analysis. **(C)** Venn diagram illustrating approach to comparing gene ontology pathway analysis between human LA and EHTs. **(D)** Selected results from gene ontology analysis using overlapping DE proteins in the Venn diagram in panel C. Gene Ontology pathway analysis accomplished using DAVID. Mass spectrometry result analysis via Proteome Discoverer (Thermo Scientific)

We next sought to determine whether the EHT model of AF also recapitulated the proteomic remodeling observed in the human samples. We performed mass spectrometry analysis on the EHT groups (Fig. 5A, **Supp. Table 3**) and subsequently, GO analysis on the DE proteins between Isometric (AF) and Cyclic (control) groups. Here, we found several overlapping pathways with the human atrial data, including multiple sarcomere-associated pathways (Fig. 5B). The data indicated a (trending) increase in β-MHC in the AF model group (**Fig. S6**), thus we manually included these genes in the DE list of genes used in subsequent analysis.

We then directly compared the DE proteins between human AF (LA) and EHT Isometric strain. As before, we identified many overlapping proteins (Fig. 5C) as expected. Pathway analysis on these proteins (**Supp. Table 3**) highlighted, among others, muscle cell differentiation and adult heart development as shared (pink, Fig. 1B; 5D). Together, these data suggest hiPSC-aCMs exposed to isometric strain model some of the functional and proteomic sarcomere remodeling observed in human AF. This may indicate that the lack of shortening in AF atrial myocytes initiates atrial sarcomere remodeling *in vivo*.

### Sarcomere Remodeling is Likely Not Mechanically Compensatory

Finally, we investigated whole organ consequences of the AF-triggered sarcomere remodeling we observed in human atrial tissue, namely increased contractility. To address this, we employed a molecular-to-organ level integrated computer simulation of a non-failing patient with AF. The model was created using the patient-specific anatomy of a 74-year-old male. Ventricular myofiber directions were assigned based on the method of Bayer et al (42) and atrial fiber directions were mapped from the Labarthe atlas (43, 44). The model was used to simulate the cardiac cycle under conditions of normal atrial active tension (100 kPa) and conditions of increased atrial contractility (up to 200 kPa) reflecting our experimental observations in AF atrial tissue.

**Figure 6:**
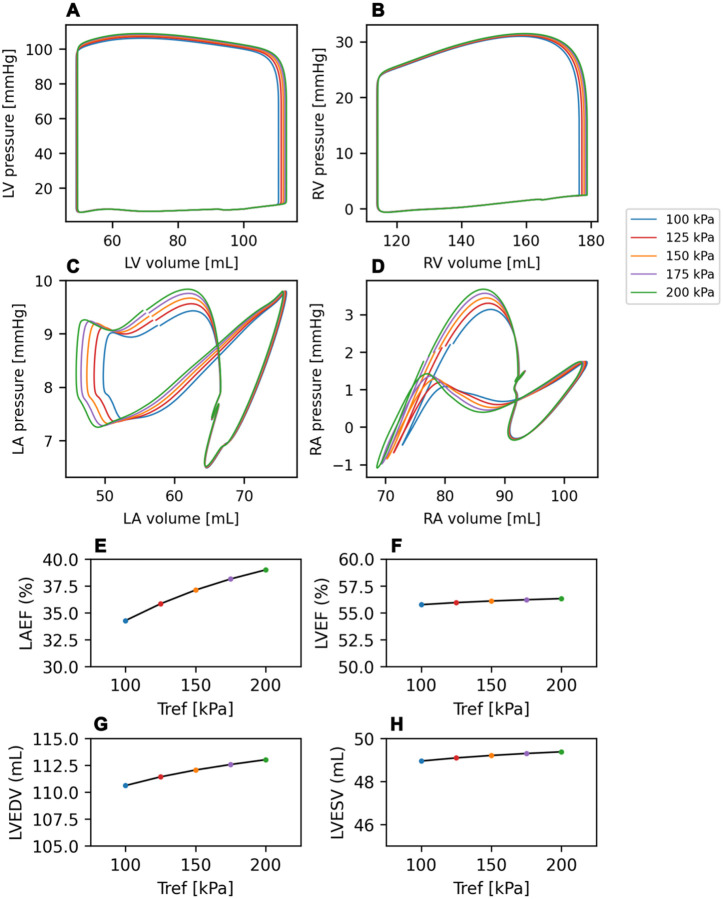
Altered Atrial Contractility in Computer Model of Non-Failing AF Patient. **(A)** Left Ventricular, **(B)** Right Ventricular, **(C)** Left Atrial, and **(D)** Right Atrial PV loops generated from a whole heart anatomical model developed from a CT scan of a 74-year-old male patient with AF (and without HF), coupled to electrical activation, mechanics, and material properties models, in which atrial contractility was altered to simulate the data in Figure 2. **(E-H)** Calculated parameters from the PV loops for left atrial ejection fraction (LAEF, panel E), Left ventricular EF (LVEF, panel F), LV end-diastolic volume (LVEDV, panel G), and LV end-systolic volume (LVESV, panel H).

Pressure-volume loops are shown for each of the atrial contractility levels simulated, for the Left Ventricle, Right Ventricle, Left Atrium, and Right Atrium (Fig. 6A–D, respectively). The simulation showed that a doubling of atrial contractility (100 to 200 kPa) resulted in a 14% increase in left atrial ejection fraction (LAEF, 34.3% at 100 kPa; LAEF, 39.0% at 200 kPa, Fig. 6E), while only a modest (<2%) effect on LVEF from a small increase in LV filling (Fig. 6F–H). Thus, though the increase in atrial sarcomere contractility is substantial, it results in relatively small changes in chamber level function in a simulated AF patient.

## Discussion

The prevalence of AF continues to increase, and therapeutic options are needed to combat this growing health concern. While much work has been done to understand the mechanisms of AF, including dysregulation of the ECM, ion channels, ROS, immune cells, metabolic pathways, and others (14, 45–49), here we hypothesized critical pathways have been overlooked. To address this hypothesis, we performed high resolution mass spectrometry on left atrial main wall tissue from non-failing patients with no evidence of valve disease, including those in normal SR and those with a reported history of paroxysmal atrial fibrillation. This analysis revealed altered signaling pathways reinforcing the currently appreciated pathobiology of AF, but also demonstrated significant dysregulation of contractile pathways involving the sarcomere, and specifically a loss of atrial isoforms. We further found that loss of atrial identify was broadly applicable to the proteome in AF. These two novel insights into the mechanisms of AF, sarcomere remodeling and loss of atrial identity, represent possible druggable targets in this growing disease.

Contractile GO pathways were dysregulated in the AF vs SR tissue and in our EHT model of AF. These expression changes were identified to be more ‘ventricular like’; indeed, “ventricular tissue morphogenesis” was among dysregulated GO pathways. Among the proteins contributing to the changes in sarcomere organization pathways was β-MHC, the molecular motor of the sarcomere which is typically lowly expressed in atria and highly expressed in ventricle (33). This finding agrees with prior research (19) which also found high β-MHC in the atrial appendage of AF patients compared to SR controls. The β-MHC isoform is stronger, slower, and more energy efficient than the α-MHC isoform (39, 50) which is typically the predominant isoform in the atria. We hypothesize that both the unexpected substantial functional increase in T_max_ in AF atrial cardiomyocytes and increased Peak Force in the EHT model of AF were due, at least in part, to this shift in myosin isoforms.

The slower kinetics of β-MHC was reflected in the slowed force production and relaxation we observed in our EHT model of AF. *In vivo*, the slower kinetics of the β-MHC isoform could result in slowed contraction that would create mechanical re-entry loops that contribute to the AF substrate. Furthermore, aside from development (51), adult, mammalian sarcomeres typically consist of one predominant myosin isoform (33, 51) –nota heterogenous population like the 50/50 split we observed here in AF. It is possible that this mechanical heterogeneity would worsen atrial contractile efficiency or even alter the sarcomere’s calcium buffering kinetics, impacting the known EC-coupling defects in AF.

β-Myosin is more energetically efficient than α-Myosin, so the switch from α to β-Myosin expression (predominant) could be an adaptation toward energetic relief for LA myocytes, as AF results in metabolic stress (45, 46), so increasing the ATP-efficient, stronger myosin isoform may assist in reducing metabolic load. However, simulations suggest that the switch to β-isoform produces only a meagre benefit to ventricular function, at the cost of placing much greater contraction stress on the LA. Hence, in a global sense the shift in myosin isoform in response to AF does not appear to be an effective adaptation. By placing added mechanical stress on atrial myocytes, the isoform switch may in fact be maladaptive.

Beyond myosin, we also observed changes in titin isoforms, ventricular regulatory light chain, myotilin (a z-disc α-actinin interacting protein (52)), troponin I, tropomyosin (Tm) alpha 3 chain, and Myosin-Binding Protein H-Like (MYBP-HL, an atrial specific protein that is incorporated into the thick filaments of atrial myocytes). Interestingly, we did not detect an increase in cMyBPC expression which is thought to be in stoichiometric balance with MyBPHL (53).

The functional consequences of this proteomic remodeling was increased sarcomere contractility in the atria. It is important to note, however, that the skinned myocyte assay utilized here assesses the contractile function of the sarcomere in isolation, as both calcium and ATP are provided exogenously. As calcium handling and mitochondrial function (11, 54) are known to be dysregulated in AF (and also altered in our MS analysis), this finding does not necessarily mean an intact cardiomyocyte will contract more strongly *in vivo*. Indeed, these changes in sarcomere function may be intended to compensate for some of these other pathological mechanisms in the cardiomyocyte.

The AF left ventricular cardiomyocytes exhibited a subtle increase in calcium sensitivity, which can be explained by our observed decrease in TnI Ser23/24 phosphorylation. These sites are targets of PKA and when phosphorylated result in a decrease in calcium sensitivity to speed relaxation at higher heart rates. Thus, decreased TnI phosphorylation likely results from decreased ventricular PKA activity, possibly from beta blockers, frequently prescribed to AF patients (55). Another possibility is that ventricular β-adrenergic activity is withdrawn as a compensatory response to the elevated ventricular rate (from the bombardment of the AV note by rapid atrial depolarizations), or to compensate for depressed calcium transient that has been observed in the ventricles of non-failing AF patients (56).

While there has been very little attention paid to the sarcomere in AF, a few studies corroborate and support an underappreciated role. Recent evidence shows that sarcomeric gene variants are directly associated with increased risk of developing AF, including TTN, MYH6, SYNPO2L, and MYL4 (20, 21, 25, 57–59). The risk was independent of cardiomyopathy, so this is not merely a ventricular disease that causes a backup of pressure into the atria causing remodeling and AF.

Furthermore, other groups have observed some of the protein changes reported here (β-MHC, titin isoforms, troponin) in AF and AF-like phenotypes in animal models (19, 20, 22, 23, 25, 60, 61), supporting our unbiased approach here. Importantly, Belus et al. examined function in myofibrils from atrial appendages and found increased β-MHC and titin N2BA expression (19), as we have. However, they observed a decrease in T_max_ associated with AF, as opposed to an increase. One possible explanation is that Belus et. al. utilized LAA tissue which, as previously described, is a vestigial tissue frequently excised during surgery due to clotting risks (62, 63). However, though more readily available, there are key differences between the LAA and the atrial main wall. The LAA is located within the pericardium, and has elevated contractility and increased shortening relative to the LA main wall (64). These differences were a motivating factor in using the LA main wall here.

We also found that over one third of all proteins altered in human AF atria overlapped with DE proteins comparing human atria versus ventricles. This strongly indicated an overall atrial loss of identity, rather than a sarcomere-specific remodeling. Similar to how chronic stress causes re-activation of the fetal gene program in the ventricles (65), it appears AF alters programs associated with chamber development or identity. Furthermore, we found that “ventricular tissue morphogenesis” and “tissue development” were significantly dysregulated GO pathways. Whether this sort of remodeling similarly occurring in other forms of atrial specific stress, i.e. atrial myopathies, is not currently known.

The most likely explanation for this observed atrial-to-ventricular shift with AF is dysregulation of upstream transcription factor(s). The probable candidate transcription factor would either be responsible for chamber identity or a factor known to target the proteins we observed altered. The leading candidate would be both. T-Box Transcription Factor 5 (TBX5) is critical for determining ventricular versus atrial cell fate (15, 66) and its expression has been linked to the expression levels of multiple genes for proteins we found differentially expressed in AF, including MYH6 and 7; Titin, MYL2, TNNI3, and TPM 1 and 2 (66–68). Furthermore, TBX5 has been found to have increased expression in AF (15), and when mutated it results in increased susceptibility to AF. Thus, TBX5 seems highly likely to contribute to the sarcomere remodeling and loss of atrial identity we observed in AF.

There are other possible transcription factors that could explain the phenotype, either on their own or in combination with TBX5. COUP-TFII and HEY2 are two transcription factors also known to contribute to atrial vs ventricular identity (69, 70), with HEY2 being a secondary target of COUP-TFII and both targeting genes such as myosin light chains.

Our atrial hiPSC-CM EHT (71) model of mechanical dysfunction associated with AF was able to reproduce the increased contractility, sarcomere protein remodeling, and altered developmental signaling we observed in human atrial tissue. We acknowledge that tissue-engineered cardiac muscle constructs are relatively immature and do not display all the hallmarks of adult myocardium. Despite these limitations, our functional EHT data agree with our human LA myocyte preparations. Additionally, our EHT model intentionally did not incorporate chronic tachypacing as a means of modeling AF to avoid a heart failure phenotype. Rather, the contractile changes noted here resulted simply from altering the mechanical milieu of the atrial EHTs. While there may be additional factors leading to our observations in human tissue, this EHT data suggests that perhaps the sarcomere remodeling is secondary to the mechanical alterations seen in AF, namely a loss of atrial kick and nearly isometric contractions. On the other hand, evidence that altered TBX5 signaling can increase AF susceptibility supports a hypothesis of loss of atrial identity and molecular remodeling resulting in AF. Thus, it remains unclear whether the signatures we have observed here are caused by AF or contribute AF development. However, a known maxim is that AF begets AF (72); these observed changes could be both a consequence and a cause of AF.

Regardless, our data suggests the sarcomere functional changes do not lead to an organ level mechanical benefit. While our model simulation did predict a modest increase in atrial ejection fraction, it was much smaller than the increase in cellular contractility observed. Along with the other likely pro-pathologic effects of proteomic cellular remodeling (isoform heterogeneity, AF associated loss of atrial identity), these modest global changes suggest the observed molecular changes are not a beneficial compensatory effect that should be encouraged/supported by therapeutic intervention, but rather maladaptive and should be prevented. Indeed, inhibiting this pathway (whether it is pathogenic or a secondary consequence) appears that it might be clinically beneficial. Targeting the transcription factors may be a useful approach, although this would require substantial care, as modulating upstream transcription factors has the potential for significant broad off-target effects. Luckily, there is now a toolbox of sarcomere targeting compounds either in development or approved for clinical use (35, 73, 74) that can be investigated in the setting of AF to determine the efficacy of targeting the sarcomere mechanical remodeling we have observed here.

There is already some evidence that targeting the sarcomere in this context may be beneficial. Propofol is an anesthetic agent used in cardiovascular surgery and is known to convert arrhythmias to SR (75). By what mechanism this occurs is not known, however it has been recently found that propofol can bind to the Ryanodine Receptor in skeletal muscle (76), and it directly interacts with myosin, actin, and myosin light chain protein (77) and induces a decrease in T_max_ (63, 77, 78)--the same parameter we saw increased in AF. Thus, propofol’s (and isoflurane’s) ability to resolve arrhythmias like AF may result from sarcomere-targeting effects that reverse functional consequences of AF that we observed here.

The findings presented in this study revealed novel cellular pathways involved in AF in the main atrial wall without the confounding effects of other cardiovascular diseases. Specifically, we observed (1) functional sarcomere remodeling (2) proteomic sarcomere remodeling from atrial isoforms to ventricular isoforms, and (3) broad proteomic signature of a loss of atrial identity. Despite substantial effort to address the growing population of patients with atrial fibrillation, new therapeutic strategies are necessary. The unbiased proteomic analysis in human tissue has uncovered a previously underappreciated role for the contractile apparatus in AF and revealed multiple potentially targetable pathways for future study.

## Methods

### Human Heart Tissue Procurement

Left ventricle (LV), left atria (LA), and right atria (RA) were obtained via the Loyola Cardiovascular Research Institute Biorepository. Tissue from nonfailing (NF) (no history of coronary artery disease or heart failure) normal sinus rhythm (SR) and atrial fibrillation (AF) rejected donor hearts was flash frozen in liquid nitrogen or isopentane. Failing hearts (heart failure; HF) were from dilated (non-ischemic) cardiomyopathy patients and were collected with informed patient consent during LVAD implantation and frozen as nonfailing tissue.

### Other Assays

Tension-calcium measurements in skinned myocytes, label-free mass spectrometry, hiPSC-CM, and engineered heart tissue experiments were performed as previously (35, 71, 79, 80).

### In Silico Atrial Fibrillation Study

The anatomical model used for this study was generated from an ECG-gated CT of a 74-year-old with AF. The model of biomechanics has been described previously (81). The Land model (82) was used to simulate active tension in the atrial and ventricular myocardium. For the baseline simulation, the reference tension in both atria (Tref) was set to 100 kPa, in agreement with previous studies (83). To investigate the effect of stronger atrial contraction on whole heart function, the simulation was repeated with Tref increased by 25%, 50%, 75% and 100%. For each simulation, the LV ejection fraction, peak pressure, end-diastolic volume and end-systolic volume were calculated, in addition to the left atrial ejection fraction.

### Statistics

Data are all presented as mean ± SEM analyzed on GraphPad Prism 9–10. Experiments were performed with 3+ biological replicates and datasets with 3+ groups were analyzed via one-way analysis of variance (ANOVA) with appropriate post-hoc tests if significance was determined. In cases of two groups, data were analyzed with a two-tailed Student’s t-test. In all cases, a *p*-value <0.05 was considered threshold for significance, though trending (*p*-value<0.1) and clarifying (*p*-value>0.1) numbers are occasionally shown above graphs.

## Figures and Tables

**Table 1.** Summary of human patient characteristics

Group	Patient #	Age (years)	Sex	Race	EF (%)
NF/SR	1	58	Male	White	N.D.
NF/SR	2	64	Male	Asian	N.D.
NF/SR	3	20	Female	White	N.D.
NF/SR	4	56	Female	Not provided	N.D.
NF/SR	5	52	Male	White	N.D.
NF/SR	6	59	Male	White	N.D.
NF/SR	7	65	Male	White	N.D.
*NF/SR group*		*53.4 ± 5.8*	*29% female*	*71.4% white*	*N/A*

NF/AF	1	70	Male	White	N.D.
NF/AF	2	60	Male	White	N.D.
NF/AF	3	50	Female	Hispanic	N.D.
*NF/AF group*		*60 ± 5.8*	*33% female*	*66.7% white*	*N/A*
P-value vs NF/SR		0.43	> 0.99	> 0.99	

HF/SR	1	37	Male	Hispanic	15
HF/SR	2	67	Female	White	30
HF/SR	3	48	Male	Black	10
HF/SR	4	64	Male	White	10
*HF/SR group*		*54 ± 7.0*	*25% female*	*50% white*	*16.3 ± 4.7*
P-value vs NF/SR		0.78	> 0.99	0.58	N/A

NF: Non-failing, SR: sinus Rhythm, AF: Atrial Fibrillation. HF: Heart Failure. N/D: Data not available.
